# Recreational drug use among individuals living with HIV in Europe: review of the prevalence, comparison with the general population and HIV guidelines recommendations

**DOI:** 10.3389/fmicb.2015.00690

**Published:** 2015-07-14

**Authors:** Noe Garin, Cesar Velasco, Jan T. De Pourcq, Belen Lopez, Maria del Mar Gutierrez, Josep M. Haro, Anna Feliu, Maria A. Mangues, Antoni Trilla

**Affiliations:** ^1^Pharmacy Department, Institut d'Investigacions Biomèdiques Sant Pau, Hospital de la Santa Creu i Sant Pau, Universitat Autònoma de BarcelonaBarcelona, Spain; ^2^Research and Development Unit, Parc Sanitari Sant Joan de Déu, University of BarcelonaBarcelona, Spain; ^3^Centro de Investigación Biomédica en Red de Salud Mental, Instituto de Salud Carlos IIIMadrid, Spain; ^4^Department of Preventive Medicine and Epidemiology, Hospital Clinic-IDIBAPS, University of BarcelonaBarcelona, Spain; ^5^ISGlobal, Barcelona Centre for International Health Research (CRESIB), Hospital Clínic - Universitat de BarcelonaBarcelona, Spain; ^6^Pharmacy Department, Hospital del Mar - Parc de Salut MarBarcelona, Spain; ^7^Grup de Recerca en HIV i AIDS-IR, Hospital de la Santa Creu i Sant PauBarcelona, Spain

**Keywords:** recreational drugs, HIV, prevalence, guidelines, recommendations, interactions, medication adherence, transmission risk

## Abstract

**Background:** Adherence problems, interactions and higher rate of risk activities have been observed in HIV individuals using recreational drugs. Our aim was to describe recreational drug use in both HIV individuals and general population in Europe, and to assess at what extent HIV guidelines address this issue.

**Methods:** Data on recreational drug use across Europe were obtained from the European Monitoring Centre for Drugs and Drug Addiction for the general population, and through Pubmed search. for HIV patients. We assessed the incorporation of recreational drug issues in HIV treatment guidelines for the following topics: (a) recreational drugs; (b) adherence to antiretrovirals; (c) interactions; (d) transmission risk. Guidelines included: World Health Organization; European Aids Clinical Society; U.S. Department of Health and Human Services; International Antiviral Society-USA; and seven European national guidelines.

**Results:** 29 countries reported recreational drug use in general population. The highest prevalences were observed for Cannabis (i.e., 8–10% in Spain, France, and Czech Republic) followed by cocaine, amphetamines and ecstasy. The 13 studies selected in the systematic review showed a great variability in recreational drug use on the HIV population. Apart from classical recreational drugs, we found a relevant use of new drugs including sexual experience enhancers. Polydrug consumption was about 50% in some studies. Most guidelines included general information about recreational drugs, showing great variability on the inclusion of the evaluated topics. We found more specific, evidence-based recommendations on interactions, followed by medication adherence and transmission risk.

**Conclusions:** Available data on the people living with HIV suggest a higher use of recreational drugs than in the general population, which is already relevant. However, recreational drug issues should be included or addressed more thoroughly in most guidelines.

## Introduction

The human immunodeficiency virus (HIV) continues to be a major public health challenge. Since the epidemic first emerged, it has been estimated that 78 million people have been infected, with 39 million deaths due to AIDS-related complications (UNAIDS, 2014). The advent of the highly active antiretroviral therapy (HAART), which restores the immune system and decreases the risk and severity of opportunistic infections, has translated into a mortality decrease by 35% since 2005 (UNAIDS, 2014). The decrease in mortality, along with the improvement of medication's safety profile and pill burden, have led healthcare systems to move from the classical conceptualization of HIV as an acute communicable disease to a modern approach in which it is considered a chronic condition (Oni et al., 2014).

Clinical management of people living with HIV should be tailored to each individual's needs. Issues such as adherence to medicines, avoidance of drug-related toxicity, and reduction of the risk of transmission have become essential goals to ensure the best health outcomes from clinical and public health perspectives. Viswanathan et al recently reported that a minimum adherence rate of about 85% is needed to suppress RNA in 80% of patients under HAART treatment (Viswanathan et al., 2014). However, a recent meta-analysis by Ortego et al. (2011) showed that only 62% of patients with HAART achieve 90% adherence or higher, which indicates the need of new efforts in improving adherence (Ortego et al., 2011). Moreover, as HIV patients live longer, long-term adverse effects from antiretrovirals may appear, which may also lead to lower adherence (Reust, 2011; Torres and Lewis, 2014; Newville et al., 2015). Finally, transmission risk is another serious challenge since 2.1 million people became infected in 2013 (UNAIDS, 2014).

Understanding how individuals' factors impact on these concerns has become crucial in the current context. With that regard, the use of intravenous (IV) drugs has been widely studied and related to poorer HIV-related outcomes, so that specific guidelines have been developed and implemented for this subpopulation of HIV patients (World Health Organization, 2014). However, the use of recreational drugs such as smoked marijuana, inhaled cocaine or oral amphetamines in HIV patients remains understudied. The little evidence that is available on this subject highlights the relevance of recreational drug consumptions at several levels. In a systematic review, Colfax et al concluded that: interactions are likely frequent since medicines and most recreational drugs share the CYP450 metabolic pathway, adherence may be decreased, and high-risk sexual behavior occurs frequently, while more evidence is needed (Colfax and Guzman, 2006). For example, protease inhibitors are known to interact with cocaine through the CYP3A pathway resulting in an increased risk of cocaine toxicity (Kumar et al., 2015). Moreover, Marquez et al. (2009) found that problems with adherence to HAART were twice as high in methamphetamine users compared with non-users (Marquez et al., 2009). In this study, it was also found that the number of sex partners in methamphetamine users doubled compared with non-users. Not only the clinical outcomes but also the prevalence of recreational drug use in people with HIV remains unclear, so clinicians are not fully aware of the real magnitude of the problem.

Despite the lack of evidence, there seems to be a certain relationship between recreational drug consumption and poor health outcomes in HIV patients. HIV guidelines should incorporate information on recreational drugs and provide clear lines of management as evidence becomes available. In this study, our aim was to provide evidence about recreational drug use in both general and HIV populations in Europe, and to assess to what extent current HIV guidelines address this issue.

## Methods

### Use of recreational drugs in the general population

Data on recreational drug consumption across Europe were obtained from the European Monitoring Centre for Drugs and Drug Addiction (EMCDDA). Information on adults aged from 15 to 64 years was collected from national representative studies. Prevalence of recreational drug use was collected for the most widely consumed drugs: cannabis, cocaine, amphetamines, and ecstasy. For some countries, data were not available for all drugs. The complete list of countries and a summary of the methodology used in each study is available in Supplementary Material.

### Use of recreational drugs in people living with HIV

**Figure 1:**
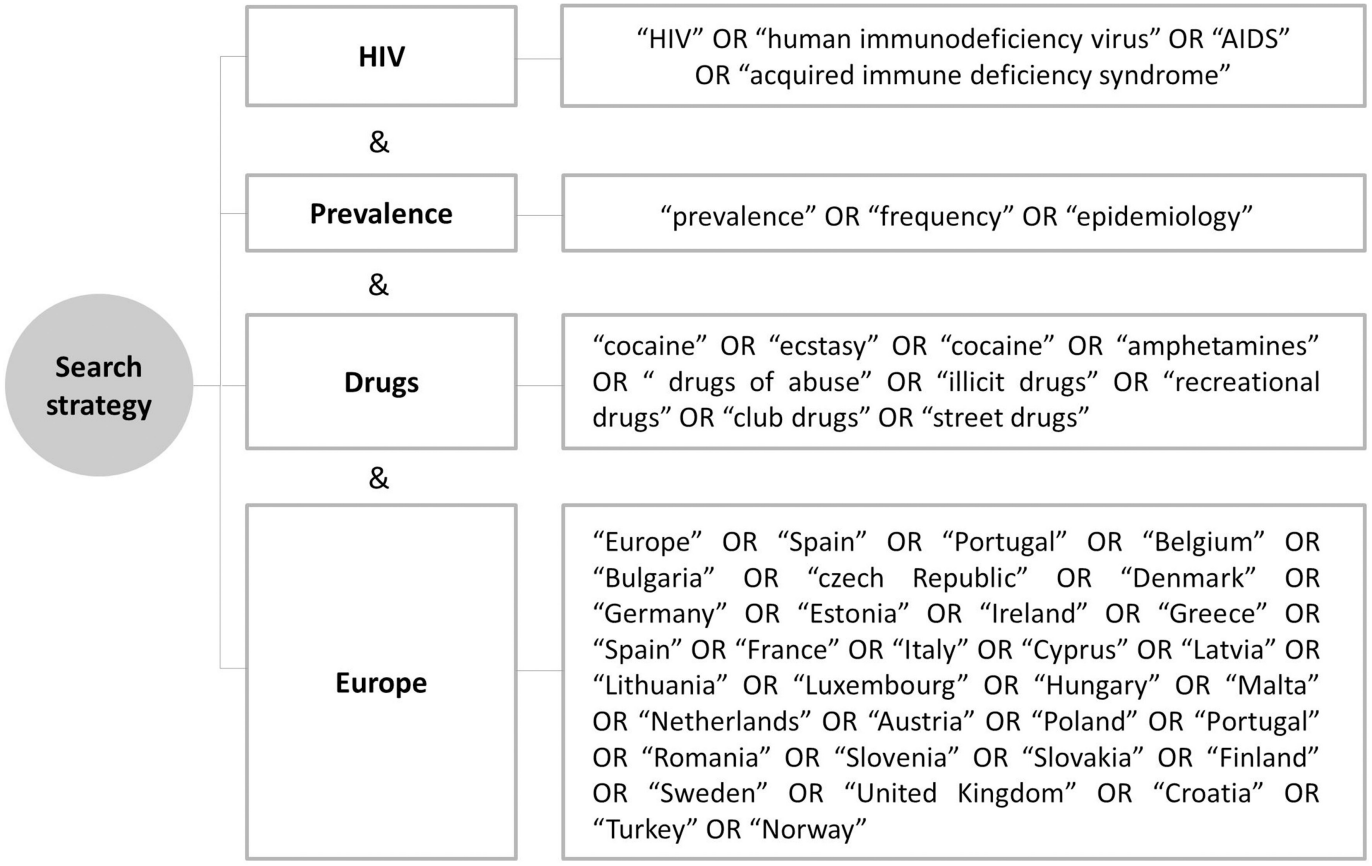
**Search strategy summary with keywords**.

A systematic review through computer searches of Pubmed was conducted on 24 December 2014 to identify original articles providing any measure of the prevalence of recreational drug use. The search strategy included four main issues related to our aim: (1) HIV; (2) recreational drugs; (3) prevalence; and (4) European setting. The strategy included MeSH terms and other terms commonly used in the literature, in the English language (Figure 1).

Search procedures were implemented to obtain the best possible evidence. Duplicates were deleted. Two reviewers screened all the articles by title/abstract and selected those that met criteria. Then, full articles were reviewed to determine whether they were suitable for inclusion. Extraction of data was performed by the reviewers. Discrepancies were analyzed by a third researcher. Non-original articles, reviews and qualitative studies were selected and reviewed when they suggested they contained any information on recreational drugs consumption. The introductions and discussions were also assessed for the selected articles to maximize the volume of articles meeting our inclusion criteria.

Selection criteria were: (a) adults over 15 years; (b) HIV population (regardless of being under treatment, specific HIV-groups, or setting); (c) outcomes from European countries. Age criteria and list of European countries were selected in line with the data provided by the EMCDDA to ensure consistency.

### Assessment of the recommendations related to recreational drugs in HIV treatment guidelines

We assessed the latest version of HIV guidelines used across Europe and also two U.S. guidelines for their relevancy in clinical practice. The final list, according to the institution or country, included: (a) World Health Organization; (b) European Aids Clinical Society (EACS); (c) U.S. Department of Health and Human Services (DHHS); (d) International Antiviral Society-USA (IAS-USA); (e) Austria-Germany; (f) France; (g) Greece; (h) Italy; (i) Portugal; (j) GESIDA-Spain; (k) British HIV Association (BHIVA).

Guidelines were assessed to identify the incorporation of recreational drug use issues by means of a search strategy, which consisted of the following keywords: cocaine, amphetamine, ecstasy, MDMA, cannabis, marijuana, heroin, LSD, ketamine, abuse, recreational, illicit, club, and “street drugs.” These keywords were adapted and translated into the languages of the selected guidelines. We then assessed the degree of incorporation of drug-related comments on three areas of interest: (a) adherence to HAART medication; (b) interactions with HAART; and (c) HIV transmission risk. The quality of recreational drug-related information in these three areas was classified into: “high,” defined as specific attention with evidence-based sections dedicated to recreational drugs and HIV issues; “medium,” defined as partial information on the topic (sufficient to understand the relevance of the topic but important concepts were not included); or “low,” defined as minimum and non-sufficient but available information concerning the specific item. E.g., mere inclusion as an example without any clear information or recommendation.

## Results

### Use of recreational drugs in the general population

**Figure 2:**
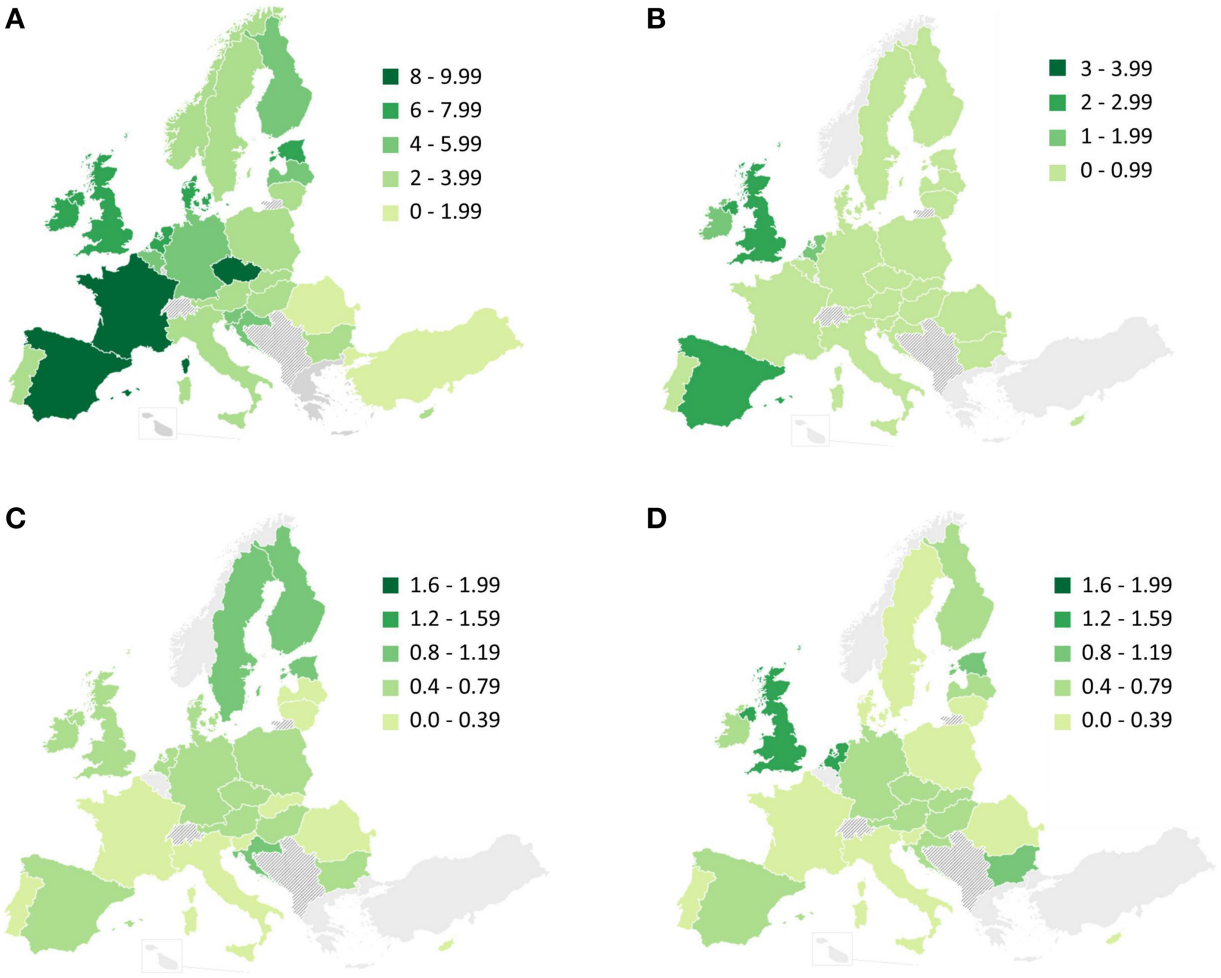
**Previous 12 month prevalence of drug use among adults (15–64 years old) for: (A) cannabis; (B) cocaine; (C) amphetamines; and (D) ecstasy**. Adapted from maps available in www.emcdda.europa.eu. Additional information regarding the studies leading to these results, such as the date when surveys were conducted, are presented in Supplementary Material.

Use of recreational drugs in the general adult population was moderate (Figure 2). Cannabis was by far the most widely consumed drug in Europe over the previous 12 months. Figures were highest (nearly 10 per cent) for Spain, France, and the Czech Republic. Other countries, such as Denmark, Estonia, Ireland, The Netherlands, and United Kingdom had a prevalence of nearly 8 per cent. Cocaine was the second most widely consumed drug in Europe, with prevalence in the previous year being above 2 per cent in Spain and the United Kingdom. Cocaine consumption was nearly 1 per cent in most other countries. The consumption of amphetamines over the previous year was relevant in some countries, especially in Nordic countries such as Denmark, Estonia, and Finland, with results near 1 per cent. Figures for ecstasy were moderate in most countries, except for Estonia, the Netherlands, and United Kingdom, where prevalence was above 1 per cent. Trends since early 1990's regarding last 12 months prevalence of drugs use in European countries are presented in Supplementary Material. Data on these trends suggest that cannabis and cocaine consumption in Europe is stable or declining, while amphetamines and ecstasy appear to be re-emerging in some areas.

### Use of recreational drugs in people under HIV treatment

We found 208 abstracts potentially fulfilling our inclusion criteria. After revision, 12 articles were found to provide data on recreational drug consumption in HIV-infected individuals. An additional article was identified and selected after reviewing the text and references of selected papers, resulting in a final list of 13 articles (Table 1).

**Table 1:** **Summary of the results (prevalence of drugs use in HIV population)**.

**Author**	**Country**	**Sample size**	**Participants'enrollment**	**Population**	**Patients under HAART (%)**	**Setting**	**Study type**	**Time reference of drug consumption**	**Prevalence of drug consumption (%)**
Allavena et al., 2014	France	1354	2012–2013	HIV-positive individuals	NA	1 HIV specialized centers	Cross-sectional	NA	Cannabis: 11.7 Cocaine: 1.7
Daskalopoulou et al., 2014	United Kingdom	2248	2011–2012	HIV-positive MSM	85.0	8 HIV specialized centers from 17 countries	Cross-sectional	Previous 3 months	Nitrites: 27.1 Cannabis: 21.2 EEM: 20.5 Cocaine: 20.2 Ketamine: 12.5 MDMA: 11.5 GHB: 9.8 Mephedrone: 7.2 Amphetamine: 3.4 Other: 8
Li and McDaid, 2014	Scotland	24 (HIV subgroup)	2011	MSM with UAI during the last 12 months	NA	17 gay commercial venues	Cross-sectional	Previous 12 months	Nitrites: 58.3 Recreational drugs: 54.2 EEM: 50
Jiménez et al., 2013	Spain	264	2011–2012	HIV-positive MSM	80.0	1 HIV specialized center	Prospective (data from baseline)	Regular use	Cocaine/Cristal methamphetamine/Alcohol: 39.4
De Ryck et al., 2013	Europe	1118	2007	HIV-positive men	80.3	17 HIV specialized centers from 17 countries	Cross-sectional	Previous 6 months	EEM: 15.1 Ecstasy/GHB (Liquid ecstasy)/Poppers: 17.6
Masiá et al., 2012	Spain	1019	2010–2011	HIV-positive individuals	65.4	13 HIV specialized centers	Cross-sectional (from a cohort study)	NA	Cocaine: 7
Dirks et al., 2012	Germany	445	2009–2010	HIV-positive MSM	NA	2 HIV specialized centers	Cross-sectional	Previous 12 months	Nitrites: 26.4 Cannabis: 19.1 EEM: 11.4 Amphetamines: 7.9 Cocaine: 3.3 Tranquilizers and bezodiazepines: 3 GHB: 2.6 Hallucinogens: 1.8 Opiates: 1.1 (including both IV and non IV use)
Schmidt et al., 2011	Germany	101	2006–2008	HIV-positive MSM	77.2	1 University (referred from HIV specialized centers across Germany).	Case-control (controls had no history of HCV infection)	Frequently or always	*Cases* (n = 34) Nasally-administered drugs (cocaine, amphetamines, ketamine): 82.4 Nitrites: 64.7 EEM: 44.1 *Controls* (n = 67) Nitrites: 53.7 Nasally-administered drugs (cocaine, amphetamines, ketamine): 52.2 EEM: 17.9
Nicholas et al., 2007	USA, Puerto Rico, Taiwan, Norway, Colombia	445	NA	HIV-positive individuals with peripheral neuropathy	77.9	Varied facilities^*^	Cross-sectional	NA	Cannabis: 11.9 Street drugs: 5.8 (no especificadas)
Peretti-Watel et al., 2006	France	2484	2003	HIV-positive individuals under HAART	100	102 HIV specialized centers	Cross-sectional	Previous 12 months	Cannabis: 26.8 Nitrites: 12.3 Ecstasy/Amphetamine/Cocaine: 5.7
Faggian et al., 2005	Italy	134	1999–2004	HIV-positive individuals using efavirenz	100	1 HIV specialized center	Retrospective	Regular use (Weekly/Daily)	Cannabis: 16.4 Cocaine: 11.2 Ecstasy: 4.5 More than one substance: 7.5
Carrieri et al., 2003	France	114	1995-2000	IV drugs users with HIV	61.4	1 HIV specialized center	Cohort (data from baseline)	Previous 6 months	Cocaine: 18.4 Psychotropic drugs: 56.1
Pérez González et al., 1999	Spain	219	1992	IV drugs users with HIV	NA	Emergency Department	Cross-sectional	Previous 30 days	Cocaine: 61 Cannabis: 53

*ACRONYMS: EEM, erection enhancing medication; GHB, gamma-Hydroxybutyric acid; HAART, highly active antiretroviral therapy; IV, intravenous, NA, data non available or not clear; MSM, men who have sex with men; UAI, unprotected anal intercourse*.

* “varied facilities” were defined as “Community-based organizations, university-based AIDS clinics, private practices, public and for-profit hospitals, residential and day care facilities and home care services.”

We found great variability in recreational drug use across studies. Cannabis and cocaine use ranged from 11.7 to 26.8% and from 1.7 to 20.2%, respectively. Nitrites emerged as one of the most widely consumed drugs, reaching 12.3–64.7%, according to the selected studies. Recreational drugs were assessed in a combined variable in some studies, with results ranging between 5.7 and 82.4%.

Methods of data collection and analysis were highly heterogeneous. The time reference to assess drug consumption varied greatly (e.g., last 3 months, last 12 months, regular use, or data not available) and the list of drugs assessed was usually brief and inconsistent across studies. With respect to the use of antiretroviral treatment, only two studies focused on individuals with HIV who were under treatment (Faggian et al., 2005; Peretti-Watel et al., 2006), while others reported treatment prevalence between 60 and 85%, or provided no information. Only one study included patients from 17 European countries (De Ryck et al., 2013). Apart from this article, data for recreational drugs were only available in six European countries.

### Quality of the recommendations related to recreational drugs in HIV treatment guidelines

Eleven guidelines were selected to be assessed. Nine of the eleven included, at some point, general comments on recreational drugs (e.g., named as illicit drugs) while only four included comments on specific drugs (e.g., cannabis, cocaine, etc.) (Table 2).

**Table 2:** **Incorporation of recreational drugs issues on principal HIV guidelines used in Europe**.

**Guideline**	**Version**	**Inclusion of recreational drugs in the Guidelines**						**Topics addressing recreational drugs in the Guidelines**			
		Recreational drugs in general^*^	**Cannabis**	**Cocaine**	**Ampheta-mines**	**Ecstasys MDMA**	Other^**^	**Transmission risk**	**Adherence to HAART**	**HAART interactions**	Other topics^***^
WHO (Worlwide)	03/2014	**YES**	NO	NO	NO	NO	NO	NE	**LOW**	NE	NE
EACS 7.1 (Europe)	11/2014	**YES**	NO	NO	NO	NO	NO	NE	**LOW**	NE	**LOW^1,2,3^**
Austria/Germany	05/2014	**YES**	NO	NO	NO	NO	NO	NE	NE	**LOW**	NE
France	11/2013	**YES**	**YES**	**YES**	**YES**	NO	**YES**	**LOW**	NE	**MEDIUM**	**LOW^6,7^**
Greece	11/2014	**YES**	NO	**YES**	**YES**	NO	**YES**	**MEDIUM**	**MEDIUM**	NE	**LOW^4,5,6,7^**
Italy	11/2013	**YES**	**YES**	**YES**	**YES**	**YES**	**YES**	**LOW**	**LOW**	**HIGH**	**LOW^2^**
Portugal	1/2015	NO	NO	NO	NO	NO	NO	NE	NE	NE	NE
GESIDA (Spain)	01/2014	**YES**	NO	NO	**YES**	NO	NO	NE	**LOW**	**LOW**	NE
BHIVA (UK)	06/2014	**YES**	NO	NO	NO	NO	NO	NE	**LOW**	**LOW**	**LOW^4^**
IAS-USA	07/2012	NO	NO	NO	NO	NO	NO	NE	NE	NE	NE
US DHHS	11/2014	**YES**	**YES**	**YES**	**YES**	**YES**	**YES**	**LOW**	**MEDIUM**	**HIGH**	**HIGH^5^**

*Inclusion of recreational drugs is categorized in yes vs. no. Topics addressing drugs are categorized in non-existent (NE), low, medium, and high*.

* *Recreational drugs in general: general mention of drugs of abuse, recreational drugs, psychotropic drugs, without specifying any of them or referring to some drugs specifically as an example of drugs in general terms*.

** *Other less consumed drugs: e.g., LSD, ketamine, nitrates, opioids, etc*.

*** *Other topics: assessment of other specific problems, named in superindex (1. Sexual dysfunction; 2. HIV-associated neurocognitive impairment; 3. Management of ALT/AST; 4. Patient support and education; 5. mental health; 6. Cardiovascular risk; 7. Broncopulmonar Risk). NOTE: In case of a general sentence such as “interactions may be plausible with recreational drugs (e.g., cocaine, cannabis) we marked YES to 3 columns (general, cocaine, cannabis)*.

As for the contents provided, four guidelines discussed habits related to transmission risk, seven presented issues regarding adherence to the HAART, and six appraised issues on drug interactions. Additionally, six guidelines incorporated comments on drugs in other sections, such as sexual dysfunction, neurocognitive impairment, mental health, and cardiovascular risk.

The degree of incorporation of drug issues in the mentioned sections was considered “low” on most occasions and “medium” in certain cases, except for the Italian and the US DHHS guidelines, which offered detailed, evidence-based information regarding interactions.

## Discussion

Our study revealed a moderate use of recreational drugs in the general population. Consumption resulted especially high for cannabis, followed at a certain distance by cocaine, amphetamine, and ecstasy. Although they do not focus on the HIV population, these results stress the magnitude of recreational drug consumption and its current trends, which to some extent may relate to drug consumption among the HIV population. Moreover, data from EMCDDA refer to an age range from 15 to 64 years but prevalence may double or triple in the young adult subgroup (15–34 years), where the risk of poly-drug use may be increased (EMCDDA, 2015). New, fashionable, geographically-specific drugs and those used by specific population groups are not covered by the data provided by the EMCDDA. Thus, caution should be taken when interpreting these results. For example, high prevalence of “mystery white powders,” with uncertain composition, has been reported in British young drug users (Global Drug Survey, 2014). Moreover, recent reports focusing on men who have sex with men (MSM) have highlighted the increase of chemsex, defined as a sexual intercourse under the influence of drugs such as mephedrone, crystal meth, or gamma-hydroxybutyric acid (GHB), taken before or during sex (The EMIS Network, 2013). Slamming, another emerging behavior that involves injecting new drugs such as mephedrone, is potentially related to an increased risk of HIV-transmission. The European Men-Who-Have-Sex-With-Men Internet Survey (EMIS) on drug use among MSM across 44 European cities showed a high prevalence of chemsex and slamming in certain MSM groups, so this is a subject to be prioritized in future studies (The EMIS Network, 2013).

To date, this is the first study aiming to summarize evidence on recreational drug consumption in the European HIV population. As expected, few studies were extracted from the original search, which mostly focused on issues other than drug consumption but reported some descriptive, valuable data on this subject. As aforementioned, we found great variability in methodology and results, so global figures on drug use cannot be extrapolated across Europe. However, results shed light on crucial aspects. First, a high prevalence of recreational drug consumption has been found in all studies. Classical drug consumption by HIV-positive people accounted for higher figures than those for the general population by the EMCDDA, reaching up to 26.8% for cannabis, 20.2% for cocaine, 11.5 for ecstasy (also referred to as MDMA) and 7.2% for amphetamines (Peretti-Watel et al., 2006; Dirks et al., 2012; Daskalopoulou et al., 2014). Higher figures in the HIV population may be biased by the relatively younger age of participants in these studies than in respondents engaged in questionnaires for the general population. Second, consumption of newer drugs is extremely frequent among people with HIV. The prevalence of ketamine, GHB (or liquid ecstasy), and mephedrone use reached 12.5, 9.8, and 7.2%, respectively in a British study (Daskalopoulou et al., 2014). Apart from their psychoactive effects, these drugs are known to be responsible for serious adverse effects and to be associated with poly-drug consumption (Winstock and Mitcheson, 2012). Third, we found a high prevalence of sex-related drug consumption, including erection enhancing medication, such as sildenafil, and nitrites (also referred as to poppers). With regard to nitrites, some studies reported prevalence figures around 60%, while sildenafil reached 20.5% (Schmidt et al., 2011; Daskalopoulou et al., 2014; Li and McDaid, 2014). Besides possible interactions with HAART, sildenafil may potentiate cardiovascular effects of nitrates, and it is therefore contraindicated (Jackson et al., 2006). Fourth, studies reporting general or multiple recreational drug consumption showed a remarkably high prevalence of their use. Results above 50% were found in two studies, underlining the need for further study on this subject since major behavioral risks may occur in these individuals (Schmidt et al., 2011; Li and McDaid, 2014).

We were unable to identify any clear patterns of use because the list of drugs to be assessed varied greatly across studies. However, there are some clarifying experiences in this regard. Semple et al assessed co-administration of methamphetamine and other drugs in HIV-positive MSM and two patterns of drug combinations were found (Semple et al., 2009). One pattern may have sexual connotations, including those drugs related to sexual performance, but others also aimed to enhance sexual pleasure, such as amphetamines and ecstasy. The second pattern would involve “party drugs,” such as cocaine, GHB, ketamine and amphetamines. Further evidence is needed to understand the motivation for co-administration and overlap in these patterns.

The high prevalence of recreational drug use in the HIV population, in line with prevalence in the general population but with its own peculiarities, highlights the need for further research. Thus, recent studies on drug-related concerns have risen, mainly focusing on high-risk sexual practices (Dirks et al., 2012; Daskalopoulou et al., 2014; Li and McDaid, 2014). Daskalopoulou et al found a high prevalence of recreational drug use in HIV-positive MSM, with increasing poly-drug use being strongly associated with increasing prevalence of condomless sex (Daskalopoulou et al., 2014). Li et al found an adjusted odds ratio of 2.75 (95% CI 1.77–4.34) of having unprotected anal intercourse with 2+ partners during the last year in men reporting use of recreational drugs (Li and McDaid, 2014). Dirks et al found similar results in terms of this association and highlighted the need for diagnostic and therapeutic strategies regarding drugs and high-risk sexual practices (Dirks et al., 2012). These studies confirm the increasing prevalence of co-occurring drug use and high-risk sexual behavior seen in daily clinical practice. In fact, it would be a specific group of HIV patients who are mainly involved in chem-sex with multiple partners, who would benefit of patient-directed management to decrease HIV risk transmission. Alternatively, providing areas with condoms but also straws and syringes for these sexual practices could be potentially useful as a preventive measure. However, evidence on this specific issue is needed to ensure its real benefit on the prevention of HIV transmission. Outside Europe, studies have shown similar associations between drugs and high-risk sexual behavior (Morin et al., 2005; Drumright et al., 2006). On this basis, it has been suggested that case history of drug consumption, including the context of use, should be performed routinely, as well as management measures according to related disorders and comorbidity (Dirks et al., 2012). Apart from sexual risk behaviors, one of the selected articles focused on the effect of recreational drugs on medication adherence (Peretti-Watel et al., 2006). In this study, some substances were associated with higher odds of low adherence to medication. Among several personal factors, recreational drug consumption has been found to impact greatly on adherence in hierarchical linear modeling (Halkitis et al., 2008). This effect may be due to temporary cognitive impairment interfering with routine activities and also deliberate loss of medication intake as a means of preventing possible interactions. With respect to interactions, none of the selected articles aimed to evaluate this issue. There is a lack of clinical evidence for most combinations of medicine/recreational drug, so most recommendations provided in pharmacological databases are based on expected theoretical interactions. Among interactions, that involving cannabis and atazanavir should be highlighted since there is the wrong impression that smoked drugs do not interact with medicines. However, one study with 64 cannabis consumers with HIV found that through levels of atazanavir were under the therapeutic range (Ma et al., 2009).

According to current evidence, we would have expected guidelines to raise awareness of all these concerns and to provide useful information to clinicians to achieve proper management of their patients. After assessing guidelines on HIV treatment used across Europe, however, it seems there is still a long way to go. Most guidelines commented on recreational drugs in general but only five mentioned specific drugs. Moreover, only four guidelines included comments on recreational drugs and high-risk sexual activities, none of them providing either in-depth information or recommendations. Given the aforementioned evidence on this topic and its importance, further information should be given in future updates. As for adherence, seven guidelines included drug-related issues. Once again, none of these remarks implied in-depth attention to the subject or clear, specific recommendations regarding recreational drugs. Regarding the impact of adherence on treatment effectiveness and the development of drug-resistance, it is clear that a structured approach is needed in future guidelines. With regard to interactions, HAART, US DHSS and the Italian Guidelines provided comprehensive, exhaustive information on specific interactions, while other guidelines treated the subject superficially or, in some cases, not at all.

Our study has several limitations. Recreational drugs market is constantly evolving while the results presented for the general population focused on classical drugs only. This may lead to unawareness of new or geographically-specific drugs, as already mentioned. Moreover, questionnaires may not reflect real consumption since drug use is considered an undisclosed issue at a social level. In this line, the response rate among drug users is expected to be lower than among non-users. Thus, underreporting may be present in some studies, especially in assessing drugs that are less accepted socially, such as the newest or strongest drugs. Additionally, we were unable to compare studies because of the great variability among studies in terms of inclusion criteria, time-references regarding drug intake (e.g., last month, last year), selection of recreational drugs, age range, and historical context. As an example, surveys conducted in gay venues may not represent either heterosexual population or MSM who do not attend these places. However, our study provides valuable information since different groups may have different behaviors regarding recreational drugs. As aforementioned, younger groups may have higher rates of drug consumption, which will especially affect comparability between figures of the general population and studies with HIV individuals. Finally, there is a possibility that certain recommendations on recreational drugs are present in specific guidelines for injecting drug users. This would explain in part the lack of information in nearly all assessed guidelines. However, since the vast majority of individuals taking recreational drugs are not classical injected-drug users, this approach may not reflect real-life situations in clinical practice.

## Conclusions

The prevalence of classical recreational drug consumption among HIV individuals in Europe is high, in line with general population trends. However, figures are greater in the HIV population, probably due to the relatively younger age of this group and sexual-related practices of certain communities. Moreover, a high proportion of HIV individuals consume new recreational drugs (ketamine, GHB, mephedrone) and drugs associated with specific sexual behaviors (erection enhancement medication and poppers). New recreational drug-use tendencies such as chemsex and slamming need to be explored and further researched at a European level due to sexual tourism. Since there is evidence on the implications of drug consumption for certain preventive and clinical outcomes, great effort should be made to address that issue from clinical and public health perspectives. Despite this need, guidelines on HIV treatment tend to omit these issues, providing no information or no clear recommendations on most occasions. Thus, implementation of specific chapters or specific guidelines focusing on issues related to recreational drugs would be recommended in the future. Guidelines would also benefit from further research on this subject to provide clear messages given the scarce research on aspects such as prevalence, adherence and interactions.

## Author contributions

NG, CV: Participated in the conception and design of the work, acquisition, analysis and interpretation of data, and drafting of the work. They also gave final approval of the version to be published and agreed to be accountable for all aspects of the work in ensuring that questions related to the accuracy or integrity of any part of the work are appropriately investigated and resolved. JP, BL: Participated in the conception of the work, acquisition of data, and critical revision of the work. They also gave final approval of the version to be published and agreed to be accountable for all aspects of the work in ensuring that questions related to the accuracy or integrity of any part of the work are appropriately investigated and resolved. MG, JH, AF, MM, AT: Participated in the conception, design, and critical revision of the work. They also gave final approval of the version to be published and agreed to be accountable for all aspects of the work in ensuring that questions related to the accuracy or integrity of any part of the work are appropriately investigated and resolved.

### Conflict of interest statement

The authors declare that the research was conducted in the absence of any commercial or financial relationships that could be construed as a potential conflict of interest.
