# Corrigendum: Recreational drug use among individuals living with HIV in Europe: review of the prevalence, comparison with the general population and HIV guidelines recommendations

**DOI:** 10.3389/fmicb.2015.01229

**Published:** 2015-11-03

**Authors:** Noe Garin, Cesar Velasco, Jan T. De Pourcq, Belen Lopez, Maria del Mar Gutierrez, Josep M. Haro, Anna Feliu, Maria A. Mangues, Antoni Trilla

**Affiliations:** ^1^Pharmacy Department, Institut d'Investigacions Biomèdiques Sant Pau, Hospital de la Santa Creu i Sant Pau, Universitat Autònoma de Barcelona Barcelona, Spain; ^2^Research and Development Unit, Parc Sanitari Sant Joan de Déu, University of Barcelona Barcelona, Spain; ^3^Centro de Investigación Biomédica en Red de Salud Mental, Instituto de Salud Carlos III Madrid, Spain; ^4^Department of Preventive Medicine and Epidemiology, Hospital Clinic-IDIBAPS, University of Barcelona Barcelona, Spain; ^5^ISGlobal, Barcelona Centre for International Health Research (CRESIB), Hospital Clínic - Universitat de Barcelona Barcelona, Spain; ^6^Pharmacy Department, Hospital del Mar - Parc de Salut Mar Barcelona, Spain; ^7^Grup de Recerca en HIV i AIDS-IR, Hospital de la Santa Creu i Sant Pau Barcelona, Spain

**Keywords:** recreational drugs, HIV, prevalence, guidelines, recommendations, interactions, medication adherence, transmission risk

Page 7, Discussion Section.

**1**. “Moreover, recent reports focusing on men who have sex with men (MSM) have highlighted the increase of chemsex, defined as a sexual intercourse under the influence of drugs such as mephedrone, crystal meth, or gamma-hydroxybutyric acid (GHB), taken before or during sex (The EMIS Network, 2013).” (Right, page 7, line 25).

**correct to** “Moreover, recent reports focusing on men who have sex with men (MSM) showed that 6% consumed drugs typically used at sex parties in the last four weeks (The EMIS Network, 2013).”

**2**. “The European Men-Who-Have-Sex-With-Men Internet Survey (EMIS) on drug use among MSM across 44 European cities showed a high prevalence of chemsex and slamming in certain MSM groups, so this is a subject to be prioritized in future studies (The EMIS Network, 2013).” (Right, page 7, line 33).

**correct to** “The European Men-Who-Have-Sex-With-Men Internet Survey (EMIS) showed that MSM who reported recent drug use also hinted at reduced control over sex and sexual risk, and reported more UAI in the same period (The EMIS Network, 2013), so this is a subject to be prioritized in future studies.”

## Conflict of interest statement

The authors declare that the research was conducted in the absence of any commercial or financial relationships that could be construed as a potential conflict of interest.

