# Supplementary material for: Recreational drug use among individuals living with HIV in Europe: review of the prevalence, comparison with the general population and HIV guidelines recommendations
Source: Front Microbiol. 2015 Jul 14;6:690. doi: 10.3389/fmicb.2015.00690 (PMC4500990; doi:10.3389/fmicb.2015.00690)
Supplement: Supplementary file 2 [file Image1.PDF]

ANNEX 2. Recreational drugs use over time in Europe

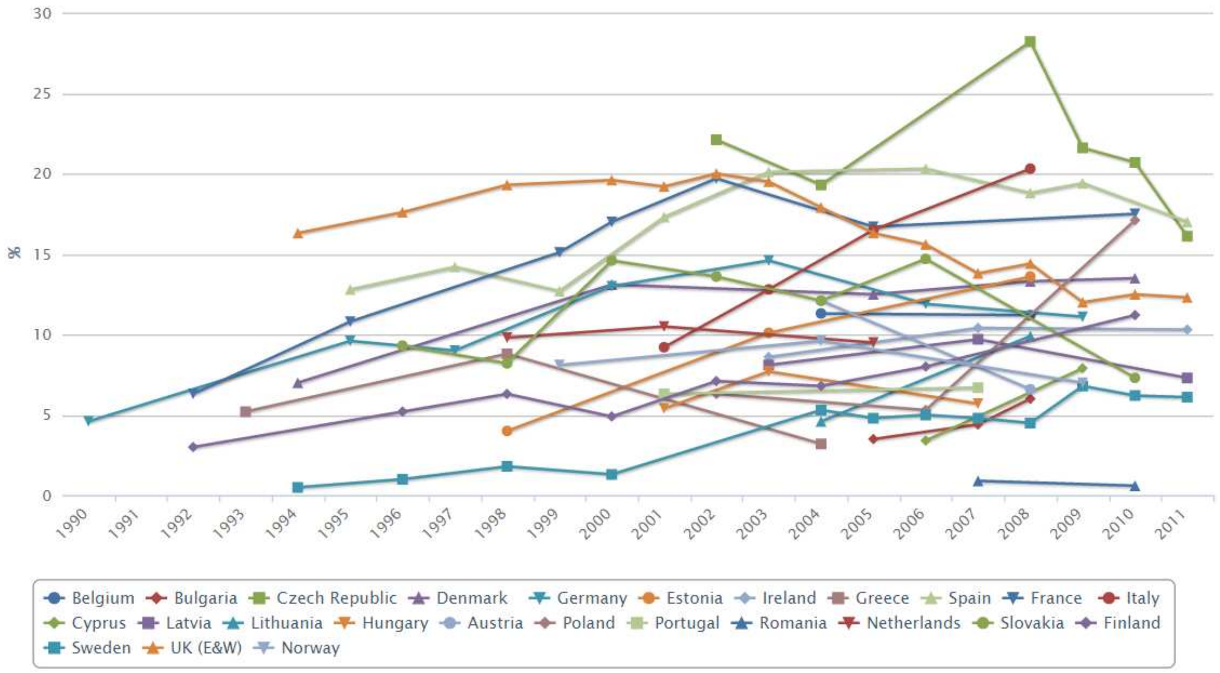

Figure 1. Trends in last 12 months prevalence of cannabis use among young adults (aged 15-34). Image taken from the EMCDDA webpage.

Original figure at: http://www.emcdda.europa.eu/stats13#display:/stats13/gpsfig4a

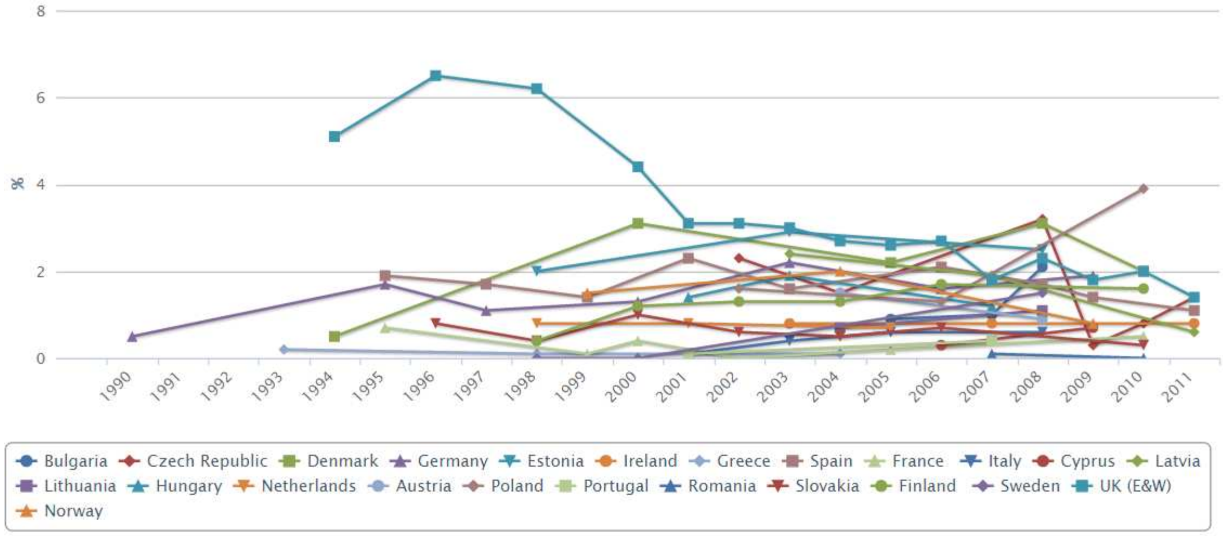

Figure 2. Trends in last 12 months prevalence of amphetamines use among young adults (aged 15-34). Image taken from the EMCDDA webpage.

Original figure at: http://www.emcdda.europa.eu/stats13#display:/stats13/gpsfig8a

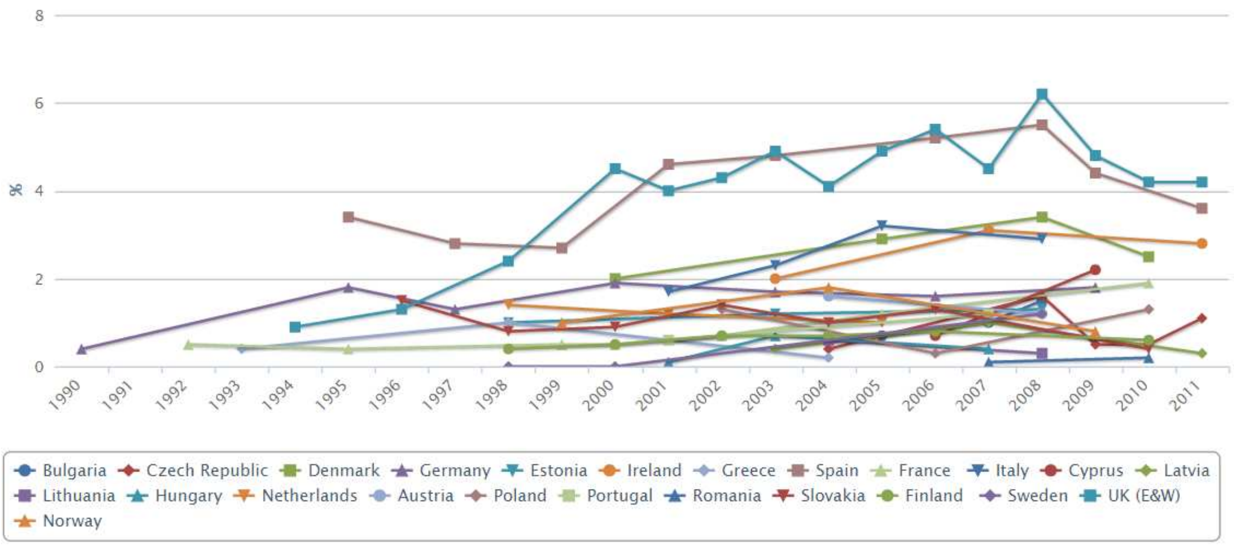

Figure 3. Trends in last 12 months prevalence of cocaine use among young adults (aged 15-34). Image taken from the EMCDDA webpage.
Original figure at: http://www.emcdda.europa.eu/stats13#display:/stats13/gpsfig14a

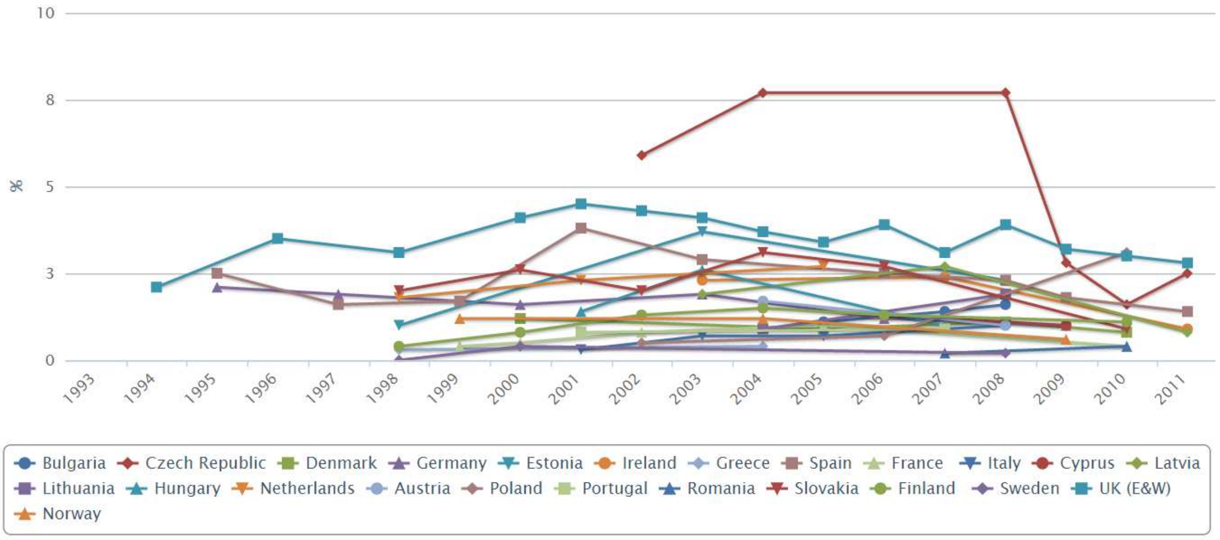

Figure 4. Trends in last 12 months prevalence of ecstasy use among young adults (aged 15-34). Image taken from the EMCDDA webpage.
Original figure at: http://www.emcdda.europa.eu/stats13#display:/stats13/gpsfig21a
